# Supplementary material for: Landscapes of Enteric Virome Signatures in Early-Weaned Piglets
Source: Microbiol Spectr. 2022 Aug 1;10(4):e01698-22. doi: 10.1128/spectrum.01698-22 (PMC9430488; doi:10.1128/spectrum.01698-22)
Supplement: Supplemental file 1 — Tables S1 to S6. Download spectrum.01698-22-s0001.pdf, PDF file, 0.1 MB [file spectrum.01698-22-s0001.pdf]

Supplemental table 1: Differential gut DNA viruses between early-weaned health and diarrhea piglets.

ID	mean (Diarrhea_piglets)	sd (Diarrhea_piglets)	mean (Health_piglets)	sd (Health_piglets)	Enrichment	p value
p__Fromanvirus	174.326	262.387	14.679	19.807	Diarrhea_piglets	0.032
p__Podoviridae	65.557	151.418	0.223	0.550	Diarrhea_piglets	0.011
f__Studiervirinae	267.185	550.717	7.876	10.026	Diarrhea_piglets	0.017
f__Dhakavirus	12.246	39.063	0.000	0.000	Diarrhea_piglets	0.016
g__Acionnavirus	42.511	54.991	668.359	1562.737	Health_piglets	0.017
g__Barbavirus	32.124	68.713	16.231	42.945	Diarrhea_piglets	0.026
g__Bastillevirus	404.528	410.568	936.461	744.248	Health_piglets	0.024
g__Bertelyvirus	338.712	591.364	64.669	75.882	Diarrhea_piglets	0.039
g__Biseptimavirus	182.998	192.438	46.579	69.104	Diarrhea_piglets	0.014
g__Cheoctovirus	377.518	432.192	832.442	941.176	Health_piglets	0.033
g__Diegovirus	1763.846	2752.184	33.333	81.305	Diarrhea_piglets	0.017
g__Kojivirus	18.332	36.951	127.391	235.611	Health_piglets	0.045
g__Nickievirus	2589.274	4437.387	38.726	78.168	Diarrhea_piglets	0.000
g__Oslovirus	1643.182	2638.516	72.280	212.759	Diarrhea_piglets	0.033
g__Peduovirus	2627.324	3792.672	262.340	488.900	Diarrhea_piglets	0.008
g__Prasinovirus	125.428	133.669	13.758	18.316	Diarrhea_piglets	0.010
g__Teseptimavirus	66.410	215.796	31.844	46.961	Health_piglets	0.049
g__Tlsvirus	671.203	1081.099	96.238	27.110	Diarrhea_piglets	0.010
s__Aeromonas_phage_CC2	49.901	111.246	13.257	36.480	Diarrhea_piglets	0.030
s__Arthrobacter_phage_Wheelbite	41.250	35.663	126.640	92.884	Health_piglets	0.010
s__Aurantimonas_phage_AmM_1	144.164	327.277	3.508	4.395	Diarrhea_piglets	0.006
s__Bacillus_phage_B4	72.748	218.583	3.690	2.840	Diarrhea_piglets	0.008
s__Beihai_mantis_shrimp_virus_3	3.095	3.475	77.945	249.031	Diarrhea_piglets	0.028
s__Brochothrix_phage_A9	38.070	104.189	0.772	1.766	Diarrhea_piglets	0.018
s__Campylobacter_phage_vB_CjeM_Los1	12.827	20.710	185.630	386.023	Health_piglets	0.017
s__Clostridium_phage_c_st	976.799	1015.947	201.050	206.421	Diarrhea_piglets	0.005
s__Cronobacter_phage_vB_CsaM_GAP32	513.696	1340.230	49.291	93.876	Diarrhea_piglets	0.014
s__Cryptophlebia_leucotreta_granulovirus	4353.988	11474.052	73.700	123.408	Diarrhea_piglets	0.017
s__Ctenophore_associated_circular_virus_4	493.936	542.944	106.878	161.053	Diarrhea_piglets	0.012
s__Enterococcus_phage_IME_EF3	67.950	72.993	17.292	20.882	Diarrhea_piglets	0.028
s__Enterococcus_phage_nattely	26.673	62.814	1.169	1.924	Diarrhea_piglets	0.008
s__Escherichia_phage_500465_1	2596.345	3785.068	206.673	347.551	Diarrhea_piglets	0.007
s__Escherichia_phage_ArgO145	1641.106	2635.804	35.679	86.420	Diarrhea_piglets	0.039
s__Escherichia_phage_nepoznato	166.226	241.143	16.647	22.134	Diarrhea_piglets	0.008
s__Escherichia_phage_phiLLS	22.724	23.868	208.061	487.635	Health_piglets	0.017
s__Escherichia_virus_TLS	671.203	1081.099	96.238	27.110	Diarrhea_piglets	0.010
s__Geobacillus_virus_E3	262.251	435.075	46.039	87.797	Diarrhea_piglets	0.020
s__Lactococcus_phage_28201	80.800	139.431	2.533	5.034	Diarrhea_piglets	0.019
s__Propionibacterium_phage_PHL301M00	211.933	331.146	30.746	23.233	Diarrhea_piglets	0.006

s__Rheinheimera_phage_vB_RspM_Barba18A	32.124	68.713	16.231	42.945	Diarrhea_piglets	0.026
s__Rhizobium_phage_RHEph10	61.816	107.213	2.772	4.038	Diarrhea_piglets	0.045
s__Shigella_phage_75/02_Stx	1705.364	2718.626	33.191	81.313	Diarrhea_piglets	0.017
s__Staphylococcus_phage_6ec	268.488	452.910	10.264	33.402	Diarrhea_piglets	0.001
s__Staphylococcus_phage_G15	52.125	150.414	3.284	7.301	Diarrhea_piglets	0.041
s__Staphylococcus_phage_P68	3.554	11.722	41.256	73.179	Health_piglets	0.026
s__Staphylococcus_phage_S25_4	0.140	0.251	140.189	483.423	Health_piglets	0.036
s__Staphylococcus_virus_108PVL	182.998	192.438	46.579	69.104	Diarrhea_piglets	0.014
s__Streptomyces_phage_Wofford	910.878	3145.902	0.767	0.674	Diarrhea_piglets	0.006
s__Synechococcus_phage_ACG_2014c	42.511	54.991	668.359	1562.737	Health_piglets	0.017
s__Synechococcus_phage_S_CAM9	18.190	39.179	215.074	528.113	Health_piglets	0.017
s__Vibrio_phage_phiVC8	201.613	379.591	71.065	112.479	Diarrhea_piglets	0.045
s__Yersinia_phage_phiR1_37	589.878	657.068	165.280	242.411	Diarrhea_piglets	0.024
s__crAssphage_cr106_1	130.104	193.357	17.627	42.575	Diarrhea_piglets	0.005
s__crAssphage_cr115_1	116.968	164.633	71.113	241.764	Diarrhea_piglets	0.042
s__crAssphage_cr118_1	1370.941	4231.475	63.077	119.264	Diarrhea_piglets	0.033
s__crAssphage_cr53_1	487.628	382.257	77.342	69.625	Diarrhea_piglets	0.001
s__crAssphage_cr6_1	102.022	132.522	28.076	54.522	Diarrhea_piglets	0.019
s__uncultured_crAssphage	94.392	200.772	110.705	95.688	Health_piglets	0.028

Supplemental table 2: Differential gut RNA viruses between early-weaned health and diarrhea piglets.

ID	mean (Diarrhea_piglets)	sd (Diarrhea_piglets)	mean (Health_piglets)	sd (Health_piglets)	Enrichment	p value
p__Artverviricota	0.000	0.000	5.133	3.938	Health_piglets	0.001
p__Kitrinoviricota	3.385	1.597	0.871	1.205	Diarrhea_piglets	0.002
c__Alsuviricetes	3.385	1.597	0.871	1.205	Diarrhea_piglets	0.002
c__Revtraviricetes	0.000	0.000	5.133	3.938	Health_piglets	0.001
o__Ortervirales	0.000	0.000	5.133	3.938	Health_piglets	0.001
o__Tymovirales	3.385	1.597	0.871	1.205	Diarrhea_piglets	0.002
f__Betaflexiviridae	3.385	1.597	0.871	1.205	Diarrhea_piglets	0.002
f__Picornaviridae	46292.001	73229.029	15189.746	8078.780	Diarrhea_piglets	0.007
f__Retroviridae	0.000	0.000	5.133	3.938	Health_piglets	0.001
g__Enterovirus	28048.137	47226.991	8697.500	6119.363	Diarrhea_piglets	0.028
g__Gammaretrovirus	0.000	0.000	5.133	3.938	Health_piglets	0.001
g__Trichovirus	3.385	1.597	0.871	1.205	Diarrhea_piglets	0.002
s__Apple_chlorotic_leaf_spot_virus	3.385	1.597	0.871	1.205	Diarrhea_piglets	0.002
s__Enterovirus_G	19193.724	31330.095	5225.227	2987.771	Diarrhea_piglets	0.004
s__Hubei_picorna_like_virus_36	0.980	1.161	582.058	1992.826	Health_piglets	0.004
s__Kirsten_murine_sarcoma_virus	0.000	0.000	5.133	3.938	Health_piglets	0.001

Supplemental table 3: Information on the correlation between gut DNA and DNA viruses.

node1	node2	p	r	|r|
s__Aeromonas_phage_CC2	*s__Rheinheimera_phage_vB_RspM_Barba18A*	0.000	0.715	0.715
s__Propionibacterium_phage_PHL301M00	*s__Staphylococcus_virus_108PVL*	0.000	0.760	0.760
s__Lactococcus_phage_28201	*s__Vibrio_phage_phiVC8*	0.000	0.638	0.638
s__Bacillus_phage_B4	*s__Ctenophore_associated_circular_virus_4*	0.000	0.740	0.740
s__Enterococcus_phage_nattely	*s__Escherichia_phage_nepoznato*	0.000	0.506	0.506
s__Staphylococcus_virus_108PVL	*s__Vibrio_phage_phiVC8*	0.000	0.592	0.592
s__Cronobacter_phage_vB_CsaM_GAP32	*s__Shigella_phage_75.02_Stx*	0.000	0.578	0.578
s__Cronobacter_phage_vB_CsaM_GAP32	*s__Escherichia_phage_ArgO145*	0.000	0.532	0.532
s__Escherichia_phage_500465_1	*s__Vibrio_phage_phiVC8*	0.001	0.510	0.510
s__Clostridium_phage_c_st	*s__crAssphage_cr118_1*	0.001	0.613	0.613
s__Lactococcus_phage_28201	*s__Staphylococcus_virus_108PVL*	0.001	0.510	0.510
s__Escherichia_phage_500465_1	*s__Escherichia_phage_ArgO145*	0.001	0.809	0.809
s__Escherichia_phage_500465_1	*s__Shigella_phage_75.02_Stx*	0.001	0.841	0.841
s__Clostridium_phage_c_st	*s__Ctenophore_associated_circular_virus_4*	0.001	0.751	0.751
s__Enterococcus_phage_IME_EF3	*s__Vibrio_phage_phiVC8*	0.001	0.533	0.533
s__Ctenophore_associated_circular_virus_4	*s__crAssphage_cr53_1*	0.002	0.584	0.584
s__Clostridium_phage_c_st	*s__Staphylococcus_phage_6ec*	0.002	0.568	0.568
s__Escherichia_phage_nepoznato	*s__Lactococcus_phage_28201*	0.002	0.566	0.566
s__Cronobacter_phage_vB_CsaM_GAP32	*s__Escherichia_phage_500465_1*	0.002	0.634	0.634
s__Enterococcus_phage_nattely	*s__crAssphage_cr53_1*	0.003	0.585	0.585
s__Enterococcus_phage_IME_EF3	*s__crAssphage_cr118_1*	0.003	0.630	0.630
s__Arthrobacter_phage_Wheelbite	*s__Staphylococcus_phage_S25_4*	0.005	0.581	0.581
s__Brochothrix_phage_A9	*s__crAssphage_cr53_1*	0.008	0.713	0.713
s__Staphylococcus_phage_G15	*s__crAssphage_cr53_1*	0.009	0.558	0.558
s__Clostridium_phage_c_st	*s__Cronobacter_phage_vB_CsaM_GAP32*	0.010	0.517	0.517
s__Escherichia_phage_ArgO145	*s__crAssphage_cr53_1*	0.012	0.505	0.505
s__Shigella_phage_75.02_Stx	*s__crAssphage_cr53_1*	0.012	0.559	0.559
s__Bacillus_phage_B4	*s__Clostridium_phage_c_st*	0.020	0.633	0.633
s__Enterococcus_phage_IME_EF3	*s__Lactococcus_phage_28201*	0.022	0.559	0.559
s__Escherichia_phage_nepoznato	*s__crAssphage_cr53_1*	0.031	0.524	0.524
s__Cryptophlebia_leucotreta_granulovirus	*s__crAssphage_cr115_1*	0.039	0.520	0.520
s__Aeromonas_phage_CC2	*s__Escherichia_virus_TLS*	0.047	0.530	0.530

Supplemental table 4: Information on the correlation between gut RNA and RNA viruses.

node1	node2	p	r	|r|
s__Apple_chlorotic_leaf_spot_virus	*s__Kirsten_murine_sarcoma_virus*	0.000	-0.807	0.807
s__Hubei_picorna.like_virus_36	*s__Kirsten_murine_sarcoma_virus*	0.034	0.605	0.605

Supplemental table 5: Information on the correlation between gut DNA and RNA viruses.

node1	node2	p	r	|r|
s__Kirsten_murine_sarcoma_virus	*s__crAssphage_cr53_1*	0.026	-0.496	0.496
s__Apple_chlorotic_leaf_spot_virus	*s__Arthrobacter_phage_Wheelbite*	0.046	-0.311	0.311
s__Apple_chlorotic_leaf_spot_virus	*s__Staphylococcus_virus_108PVL*	0.037	0.434	0.434
s__Apple_chlorotic_leaf_spot_virus	*s__Yersinia_phage_phiR1_37*	0.034	0.377	0.377
s__Apple_chlorotic_leaf_spot_virus	*s__Rhizobium_phage_RHEph10*	0.007	0.588	0.588
s__Enterovirus_G	*s__Lactococcus_phage_28201*	0.002	0.393	0.393
s__Enterovirus_G	*s__crAssphage_cr53_1*	0.002	0.587	0.587
s__Enterovirus_G	*s__Escherichia_phage_nepoznato*	0.000	0.455	0.455
s__Enterovirus_G	*s__Enterococcus_phage_nattely*	0.000	0.593	0.593
s__Enterovirus_G	*s__Streptomyces_phage_Wofford*	0.000	0.063	0.063

Supplemental table 6: Number of reads used for metagenomic and metatranscriptomic analyses

Sample ID	Number of reads	
	Metagenome	Metatranscriptome
Diarrhea-1	77361870	86356474
Diarrhea-2	63510136	87196242
Diarrhea-3	76007726	68791960
Diarrhea-4	74222548	73428246
Diarrhea-5	80669004	96446206
Diarrhea-6	78307546	85983314
Diarrhea-7	71439072	97430274
Diarrhea-8	94629342	91503302
Diarrhea-9	79307024	87544788
Diarrhea-10	75490974	102375574
Diarrhea-11	65324114	99825464
Diarrhea-12	72008162	83361056
Health-1	75250206	68681218
Health-2	70611344	70348814
Health-3	72052066	75153710
Health-4	71814856	72410548
Health-5	79932932	72086184
Health-6	85874646	71258682
Health-7	71361852	77050140
Health-8	86972516	83434768
Health-9	89156106	97333224
Health-10	83239840	90423058
Health-11	75788686	73760688
Health-12	98357742	73641396
